# A Four Element mm-Wave MIMO Antenna System with Wide-Band and High Isolation Characteristics for 5G Applications

**DOI:** 10.3390/mi14040776

**Published:** 2023-03-30

**Authors:** Mehr E. Munir, Saad Hassan Kiani, Huseyin Serif Savci, Mohamed Marey, Jehanzeb Khan, Hala Mostafa, Naser Ojaroudi Parchin

**Affiliations:** 1 Smart Systems Engineering Laboratory, College of Engineering, Prince Sultan University, Riyadh 11586, Saudi Arabia; 2 Electrical Engineering Department, Iqra National University, Peshawar 25000, Pakistan; 3 Electrical and Electronic Engineering Department, Istanbul Medipol University, Istanbul 34810, Turkey; 4 Department of Information Technology, College of Computer and Information Sciences, Princess Nourah bint Abdulrahman University, P.O. Box 84428, Riyadh 11671, Saudi Arabia; 5 School of Engineering and the Built Environment, Edinburgh Napier University, Edinburgh EH10 5DT, UK

**Keywords:** olympeak, linear array, MIMO system, 28 GHz, 5G

## Abstract

In this article, we propose a light weight, low profile Multiple Input Multiple Output (MIMO) antenna system for compact 5th Generation (5G) mmwave devices. Using a RO5880 substrate that is incredibly thin, the suggested antenna is made up of circular rings stacked vertically and horizontally on top of one another. The single element antenna board has dimensions of 12 × 12 × 0.254 mm${}^{3}$ while the size of the radiating element is 6 × 2 × 0.254 mm${}^{3}$ (0.56λ${}_{0}$ × 0.19λ${}_{0}$ × 0.02λ${}_{0}$). The proposed antenna showed dual band characteristics. The first resonance showed a bandwidth of 10 GHz with a starting frequency of 23 GHz to an ending frequency point of 33 GHz followed by a second resonance bandwidth of 3.25 GHz ranging from 37.75 to 41 GHz, respectively. The proposed antenna is transformed into a four element Linear array system with size of 48 × 12 × 0.254 mm${}^{3}$ (4.48λ${}_{0}$ × 1.12λ${}_{0}$ × 0.02λ${}_{0}$). The isolation levels at both resonance bands were noted to be >20 dB which shows high levels of isolation among radiating elements. The MIMO parameters such as Envelope Correlation Co-efficient (ECC), Mean Effective Gain (MEG) and Diversity Gain (DG) were derived and were found to be in satisfactory limits. The proposed MIMO system model is fabricated and through validation and testing of the prototype, the results were found to be in good agreement with simulations.

## 1. Introduction

In the modern world, wireless communication is impacting countless areas (e.g., dependable and effective remote communication, remotely automated and robot-controlled machines, machine-to-machine communication, the Internet of Things (IoT), unmanned transportation systems, smart grid concepts in power transmission and distribution, digital banking systems, smart home HDTV through effective satellite communication systems), and these technologies are providing great significance in improving human lives [1,2,3]. Antenna engineers have been working to create creative and effective solutions for the continuous and uninterrupted connection with perfect and consistent reception of the signals in light of the rapid expansion of wireless communication technologies over the past few decades [4]. Wireless service providers usually face previously unheard-of difficulties as they attempt to address a global bandwidth constraint due to the rapid growth of mobile data and the widespread usage of smart phones.

MIMO antennas are extremely beneficial in densely populated areas where Line of Sight (LOS) communication is impossible. The signal may be in-phase or out-of-phase when it arrives at the receiver via multi-path in these circumstances, causing multi-path fading. The MIMO antenna, which is coupled with a combiner to increase the mean signal-to-noise (SNR) ratio and produce diversity gain, reduces the multi-path problem. There are four types of diversity combiners: switching, equal gain combining (EGC), maximum ratio combining, and selection combining (MRC). The branch with the highest SNR is chosen at any point in time in selection combining. The signal from the branch that meets the minimal threshold value is chosen by the switched combiner. The co-phased branch signals are added by EGC. Each branch in MRC has its phase weights applied so that the result is the sum of all of the SNR ratios. MIMO antennas when initially employed, boost spatial variety in order to combat channel fading. The information is being considered in a Rayleigh fading environment, where it is sent by antennas that take various routes to independently reach receiving antenna. The highest diversity increase in this scenario is referred to as Spatial Diversity. When separate information is delivered over antennas, the data rate is increased, which is known as Spatial Multiplexing. MIMO antennas have given wireless communication a new direction and are now particularly desirable for 5G applications. Higher data rate, low latency and with better system stability are the characteristics which make 5G a potential candidate for future wireless communication systems. Designing a MIMO antenna system is a complex task since modern communication devices are desired to be compact and slim. A wide scale placement of a 5G network requires the preparation of antenna infrastructure and the execution of new technical clarifications [5,6]. The millimeter wave spectrum starting from 20 GHz to 300 GHz is well known for its multi gigabit/s transmission rate and larger bandwidth availability to meet the needs of 5G applications. The frequency bands centered at 28, 38, 60, and 73 GHz have been allocated for 5G mobile networks by the International Telecommunications Union (ITU). These frequency bands are currently unlicensed and are free to use. When using antennae for a communication system, the power is transmitted or received according to the spatial characteristics established for a network which results in determining the predefined users location. Several MIMO antenna systems have been proposed in the literature. These MIMO systems include Substrate Integrated Waveguide (SIW) based antennas, Empty Substrate Integrated Waveguide (ESIW), Air-gap Filled Antennas (AFAs), Dielectric Resonators (DRA) and planar antenna systems [7,8,9,10,11,12,13,14,15]. In [7], triband DRA is proposed for mmwave applications. The triband DRA had stacked radiating elements with semicircular slots etched on each side of top dielectric surface. The resonance bandwidth included 7.34 GHz, 4.04 GHz and 3.30 GHz at resonance frequency of 28, 33 and 38 GHz. A printed dipole four element MIMO antenna is presented in [8]. The total size of the MIMO system is 48 × 31 mm${}^{2}$ with a bandwidth offering of 5 GHz from 26 to 31 GHz. The isolation of the MIMO elements is increased up to 21 dB with the integration of a capacitive loaded loop unit cell. A four-element MIMO antenna in [9] provides resonance bandwidth of 3GHz with ECC < 0.0005 among any two radiating elements. Antenna configuration using orthogonal assembly in the mmwave spectrum has thoroughly been investigated in [10,11,12,13]. A dual band mmwave MIMO antenna system for 5G technology is presented in [10]. Additionally, the placement of the antenna elements in an array was constructed in such a way that an acceptable amount of isolation was obtained without the use of conventional decoupling structures or processes. A system made up of four radiating elements and an etched Rogers-5880 substrate having permittivity of 2.2 with a thickness of 0.508 mm was used to illustrate this. An E-shaped patch, an H-shaped slot inside a patch, and a transmission line make up each radiating element. The system has a minimum port isolation of −28 dB and resonates at two separate mmwave frequencies, namely 28 GHz and 38 GHz with 7.1 dBi at 28 GHz and 7.9 dBi at 38 GHz with average efficiency, and envelope correlation coefficient (ECC) of the system at 70%. A planar 5G MIMO antenna is presented in [11] with total size of 30 × 30 mm${}^{2}$. The four antenna elements in the proposed MIMO antenna are positioned 90${}^{\circ }$ apart. Four circle-shaped ring patches make up the transmitting component of each antenna element, and they primarily help the device to operate within the intended frequency range. Additionally, the centre of each antenna element is surrounded by four rings with circles on them, each of which is above the feed line. Minimal mutual coupling is shown by the isolation of more than −29 dB for the working band. Additionally, the suggested MIMO antenna’s peak gain and overall efficiency for the working bandwidth are 6.1 dBi and 92%, respectively. Similarly, in [12] the slot monopole antenna systems were presented with a size of 30 × 30 mm${}^{2}$. The antenna was comprised of small five circular arms connected to main circle hence resulting in form of petal flower. The antenna showed a bandwidth of 2 GHz with a central frequency of 28 GHz and peak gain of 5 dBi. In [13] a four element antenna system was presented at 38 GHz with peak gain of 5 dBi and ECC < 0.01. The DRA presented in [14] was designed in a ’plus’ shape with radiating elements directly facing each other. Similarly, ESIW and AGF antennas for mmwave have also been reported [15,16].

In [17], for upcoming 5G cellular applications, a dual-band dual-linear polarisation reflect array design is created. For this reason, a single layer unit cell containing two pairs of miniature fractal patches is made to operate in a dual-polarization mode at two separate frequencies in the Ka-band (27/32 GHz). The complete independence between the intended frequency bands and polarisations is shown by in-depth analysis of unit cell behaviour. Small unit cell sizes, minimal losses, a very simple and thin construction, and an independent optimization of the phase at each frequency and polarisation are all features of the suggested architecture. Two pairs of miniature patches are combined to form the proposed dual-band, dual-polarized reflectarray cell, which is printed on the same substrate layer. Each pair functions at a certain resonant frequency. Each pair operates at a specific resonant frequency, two linearly polarized elements are assumed, which are each rotated by 90${}^{\circ }$, thus offering a dual polarization operation mode.

Four different random array types were contrasted in [18]. Specifically, the array factor’s mean and variance calculations were derived. Hence, offering a partial statistical characterization that enabled highlighting certain key characteristics of random arrays and linking them to the elemental count and aperture of the array. In addition, additional weight was also placed on the experimental part because there was not a straightforward analytical tool that worked well enough, especially when considering the side-lobe level. For achievement purposes, Monte Carlo simulations were carried out in order to experimentally construct the side-lobe distribution as a function of the quantity of radiators and the typical distance between neighbouring radiators. The experimental results obtained showed that random arrays with the freedom to set restrictions on the minimal distance between adjacent components were achieving performance comparable to that of other techniques without such restrictions. However, the former were preferred as they can reduce the impacts of reciprocal coupling by reducing the likelihood that nearby radiators will be closer together than a predetermined minimum distance.

In order to enhance the isolation among the radiating elements, several approaches are considered. These techniques include isolating structures insertion such as neutralization lines (NL), Defected Ground Structures (DGS), Electromagnetic Bandgap Structures (EBG) and Artificial Magnetic Conductors (AMC) [19,20,21]. An antenna for mobile terminals with a wideband printed dual-antenna is examined in [19]. The dual-antenna is printed on a printed circuit board and consists of two symmetric antenna elements and three neutralization lines (NLs). The mutual coupling is reduced throughout a broad frequency spectrum by the three NLs. S-parameters, surface current distributions, and a simplified equivalent model are used to assess the three NLs’ functioning mechanisms and provide simple guidelines for optimizing them. With the insertion of isolating structures, a 6 dB enhance isolation was achieved. Similarly, in [20], a DGS was considered in order to implement effective isolation between units of a multi-input multi-output (MIMO) antenna. Specifically, a two-element antenna with coaxial feeding that operates at 5.8 GHz. The middle of the two elements created as a DGS had a zigzag groove placed into it to lessen mutual coupling between the parts. A scattering matrix, including the reflection coefficient S11 and transmission coefficient S21 between two element ports, was tested to confirm this design. The antenna’s diversity gain, envelope correlation coefficient (ECC), current distribution, and radiation pattern were all simulated and tested at the same time. The results revealed that using a DGS reduced mutual coupling by 28.8 dB, and the ECC was less than 0.02.

An innovative EBG design and a hair-pin-shaped DGS are used in combination in [21] to achieve excellent isolation between two-element MIMO antennas operating at 27.5–28.35 GHz. The suggested EBG provides a broad frequency band-gap between 26.2 and 32.03 GHz by being developed on layered dielectric substrates. In order to minimise surface wave coupling, a 2 × 3 array of the EBG is placed between two electromagnetically coupled radiating patches. A SIW feeding network and cavities are selectively integrated into the proposed antenna for boosting the radiation output and decreasing feed losses. When contrasted to an unloaded MIMO antenna, EBG exhibits an average isolation improvement of 13.9 dB within the 5G spectrum. In [22], an eight element MIMO system is presented in which the isolation is increased up to 15 dB with insertion of isolating structure. Similarly in [23] a DGs technique is used to enhance isolation up to 21 dB. In [24] an EBG induced dual band MIMO antenna is presented. With the help of PIN diode-integrated branch-lines (BLs), the isolation is increased. In [25], a two element MIMO system is presented at mm-wave band with a central frequency of 28 GHz with a parasitic element. This parasitic element is used for self-field cancellation for the MIMO configuration. In [26], the isolation among closely packed MIMO elements is increased with help of DGS slots.

This research presents a simple planar monopole MIMO antenna system arranged in linear manner. The proposed MIMO antenna exhibits dual wide-band characteristics covering two mmwave 5G alloted resonances of 28 and 38 GHz, respectively. The four element linear MIMO system satisfies the MIMO performance parameters and also gives high gain of 11 dBi. The paper is organized as follows. Section 1 covers the detailed literature review and introduction of mmwave MIMO systems. Section 2 covers the antenna design analysis and its configuration into MIMO form. Section 3 presents the results obtained from the fabricated prototype followed by the conclusion.

## 2. Antenna Design

**Figure 1:**
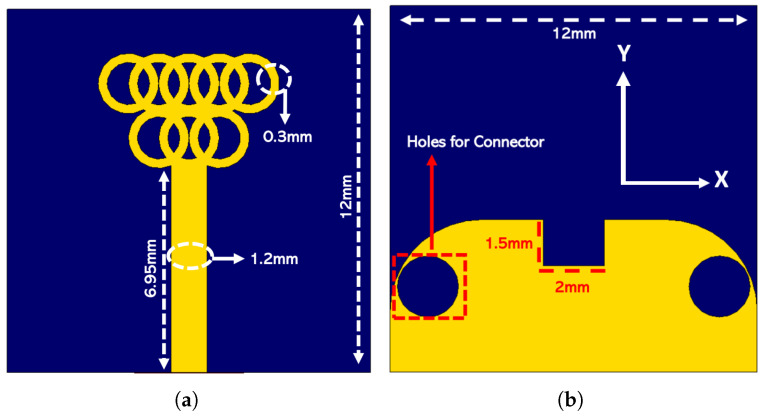
Olympeak antenna (**a**) front and (**b**) back.

**Figure 2:**
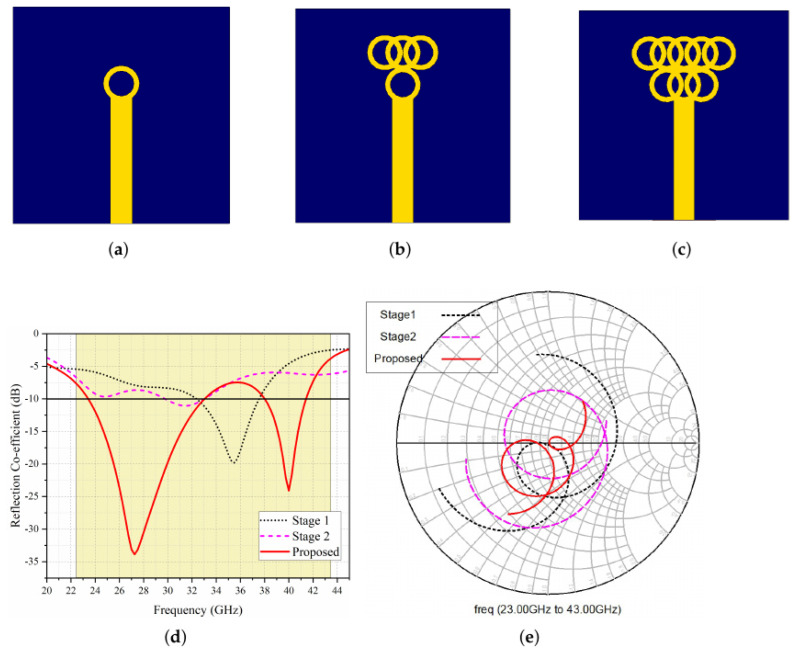
Design evolution (**a**) stage 1 (**b**) stage 2 (**c**) proposed (**d**) reflection co-efficient (**e**) Smith chart.

Figure 1 shows the proposed olympeak shape mmwave single element design. The antenna is designed on a 0.254 mm ultra-thin RO5880 substrate with a relative permittivity of 2.3. The proposed antenna comprises of ring structures stacked among each other. The circles assembled in such close assembly resembles the “Olympic” logo but with an enhanced number of circles. Hence, making a hybrid form which is named as “olympeak”. The back side of the design comprises of bent corners on each side and a square slot on the middle section which helps in tuning the antenna response to desired frequencies. The design evolution of the proposed olympeak antenna is shown in Figure 2. At the first initial stage, a feed-line with a small single circular ring was presented. In this initial stage, the frequency bandwidth of almost 4 GHz was achieved with a starting frequency of 32 GHz to 36 GHz. This design was further extended with three circular rings intersecting each other in stage 2. The intersecting rings shifted resonance frequency backwards to 30 GHz with a resonance bandwidth of only 2 GHz. The intersecting rings were increased in the proposed stage, by a total of eight rings. The intersecting rings were placed at each side of the lower circular ring and higher circular ring rows. In the proposed stage, the antenna showed a dual wideband response from 24 to 34 GHz and and 37.5 to 41.5 GHz. The s-parameter of the different stages of design evolution can be seen in Figure 2d.

The impedance matching of the proposed olympeak antenna in evolution stage is shown in Figure 2e. It is seen on the Smith chart that as the number of intersecting rings were increased, the voltage standing wave ratio improved. In the proposed design, additional resonances were introduced to enhance the operational bandwidth of the single antenna by optimizing the intersecting rings.

**Figure 3:**
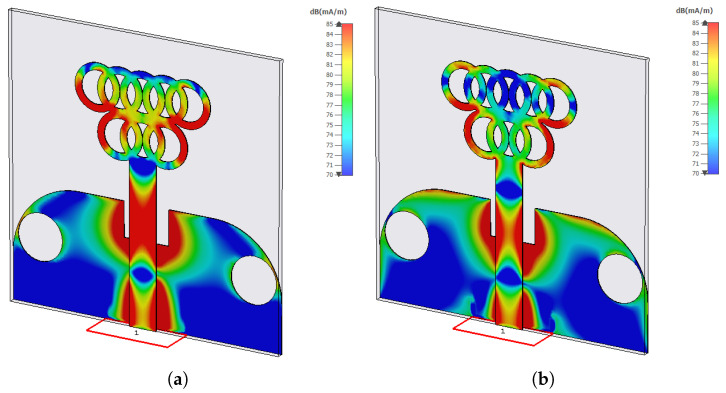
Surface currents of the olympeak antenna at a resonance frequency of (**a**) 28 GHz (**b**) 38 GHz.

The surface current patterns at two resonances of 28 and 38 GHz are shown in Figure 3. At both resonances it can be observed that the currents are focused on the outer ring edges and ground slot which circulates strong currents. The holes etched for connectors have an almost uniform current distribution.

**Figure 4:**
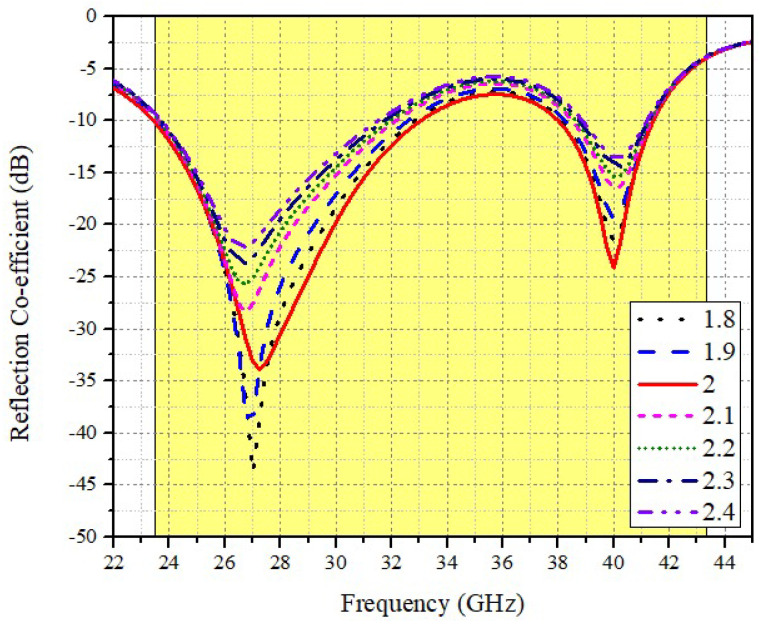
Ground slot effect on resonance response of the olympeak antenna.

The effect of the ground plane notch etched at the top mid-section of the copper ground plane is given in Figure 4. The ground slot played an important role in shifting the resonance response to desired frequency. At 1.8 mm slot width, the maximum resonance was noted at 27.5 GHz which at 1.9 mm resulted in a very sharp dip, hence giving a reflection co-efficient value of −45 dB at the same frequency. With a further gradual increase of 0.1 mm, the optimum response was noted at 2 mm. Further increasing the value resulted in a diminishing resonance response.

The radiation and total efficiency of the antenna is >82% in the operating band. The peak gain is noted to be 4.9 dBi while on 28 GHz its 4.2 dBi with radiation and total efficiency of 94 and 95%, respectively.

**Figure 5:**
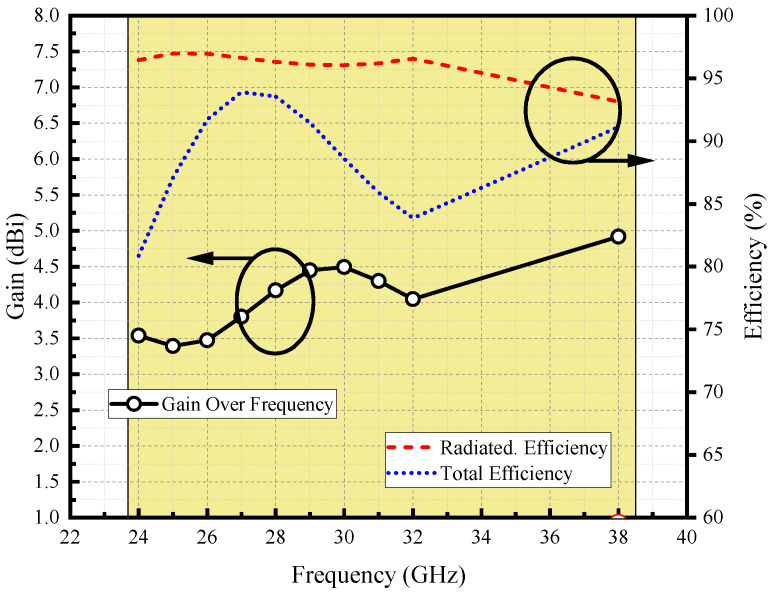
Efficiency and gain of the proposed antenna.

**Figure 6:**
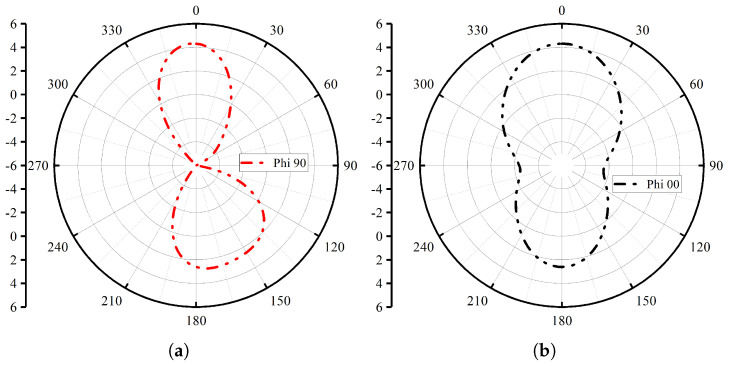
Radiation patterns at 28 GHz frequency on plane (**a**) zy and (**b**) zx.

The performance parameters of the antenna mentioned above are shown in Figure 5. The radiation pattern at two principle plane xy and zx plane is shown in Figure 6. At the zx plane, the main lobe direction is at 0${}^{0}$ with 3 dB angular width of 85${}^{0}$ while for the xy plane, the main lobe is slightly tilted to 358${}^{0}$ with a side lobe level of −1.3.

## 3. MIMO Configuration

**Figure 7:**
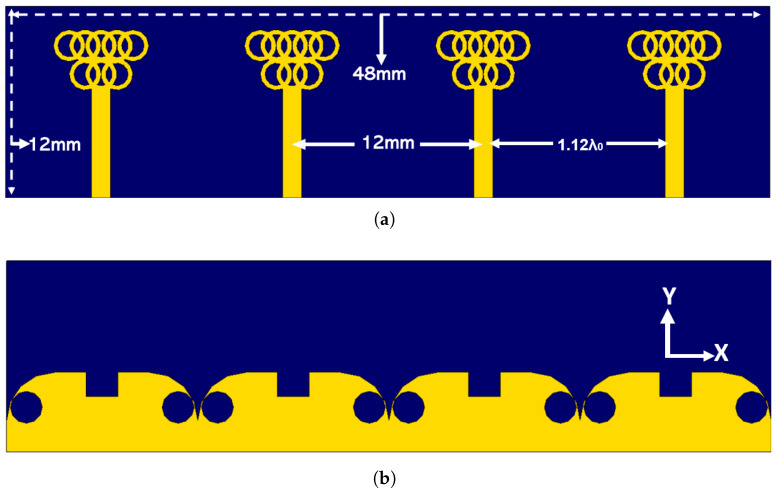
Olympeak MIMO antenna (**a**) front view (**b**) back view.

The proposed olympeak antenna is transformed into a four element MIMO antenna system. The MIMO elements are transformed into a linear element, having 12 mm distance among each element centre to centre. The distance among the MIMO elements is kept at 1.2 λ at 28 GHz resonance. The dimensions of the MIMO antenna system is noted to be 48 × 12 × 0.254 mm${}^{3}$. Figure 7 shows the proposed olympeak linear MIMO Antenna.

**Figure 8:**
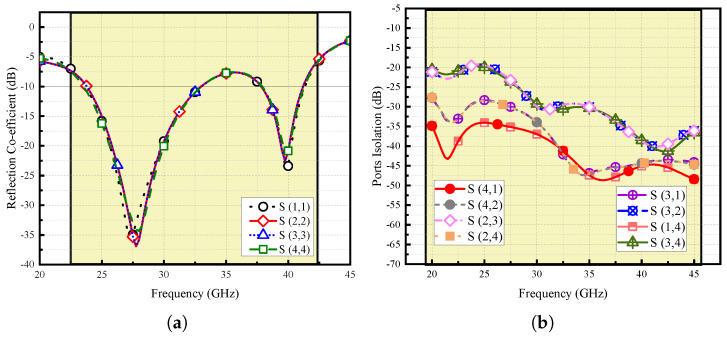
Olympeak MIMO antenna simulated s-parameters (**a**) reflection co-efficient (**b**) ports isolation.

The MIMO simulated s-parameters are shown in Figure 8. Figure 8a shows the reflection co-efficient response while the ports isolation are shown in Figure 8b. The reflection co-efficient of the MIMO antenna is the same as the single element resonance response while for isolation characteristics, the first band offers the peak coupling value of 20 dB for side-by-side elements closest to each other, and for the second resonance, the enhanced isolation characteristics of >33 dB is observed.

## 4. Prototype Development and Measurement

**Figure 9:**
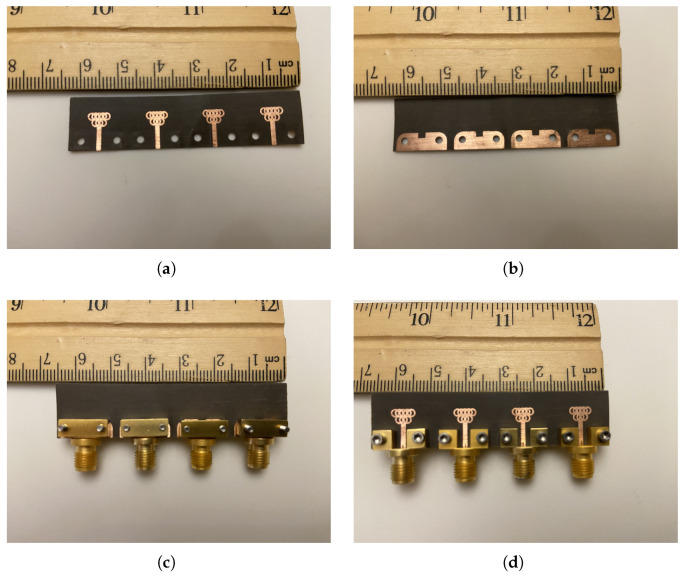
Olympeak MIMO antenna prototype (**a**) without connector front (**b**) without connector back (**c**) with connector back (**d**) with connector front.

The proposed olympeak MIMO antenna system is fabricated, and using in-house facilities, the measurements have been taken from the developed prototype. The fabricated prototype is shown in Figure 9. Figure 9a,b shows the proposed fabricated prototype model without connectors while Figure 9c,d shows the prototype with south-east connectors. In order to verify the design dimensions, a scale has been placed near the fabricated prototype.

**Figure 10:**
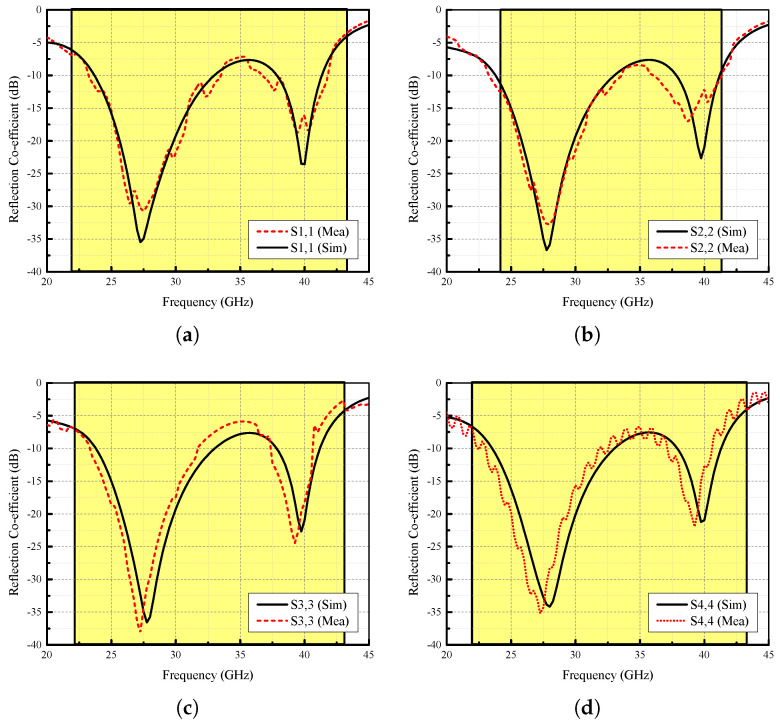

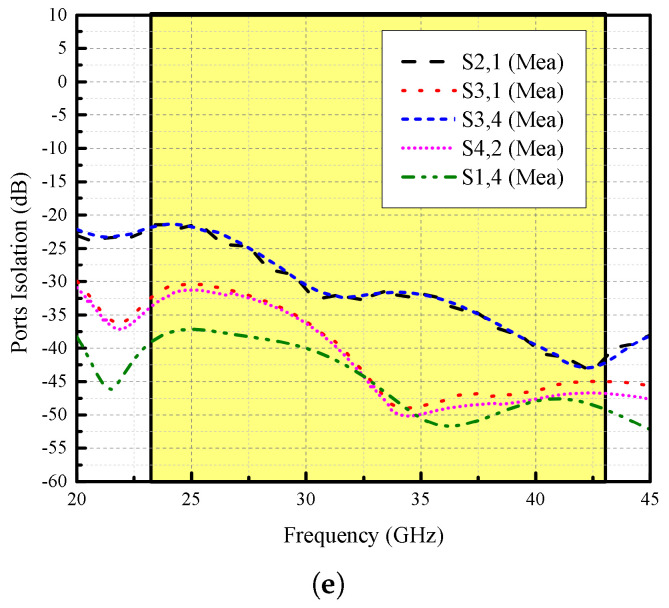
Olympeak MIMO antenna measured s-parameters (**a**) antenna 1 (**b**) antenna 2 (**c**) antenna 3 (**d**) antenna 4 (**e**) ports isolation.

The measured and simulated s-parameters are superimposed on the plots in Figure 10. The solid-black lines are from the finite difference time domain-based electromagnetic simulations, whereas the dashed red lines are from the measured data. The slight ripples in the measurements are due to drifted calibrations and bent cables.

The antenna is so thin and flexible that it requires a delicate mechanical balance to keep steady during the measurement. It is seen from the plots in Figure 10a–d that the measurement and simulated reflection coefficients are in great agreement. The slight shifts in the pole locations are due to slight bends of the substrate and also they are well within measurement uncertainty. Figure 10e shows the simulated and measured ports isolation performance between the antenna elements on the array. The slight difference between simulations and measurements can be attributed to the aforementioned calibration and bend issues. However, an isolation performance of at least 20 dB is achieved as seen in the figure. The measured isolation value is obtained as 24 dB at 28 GHz and 37 dB at 38 GHz.

**Figure 11:**
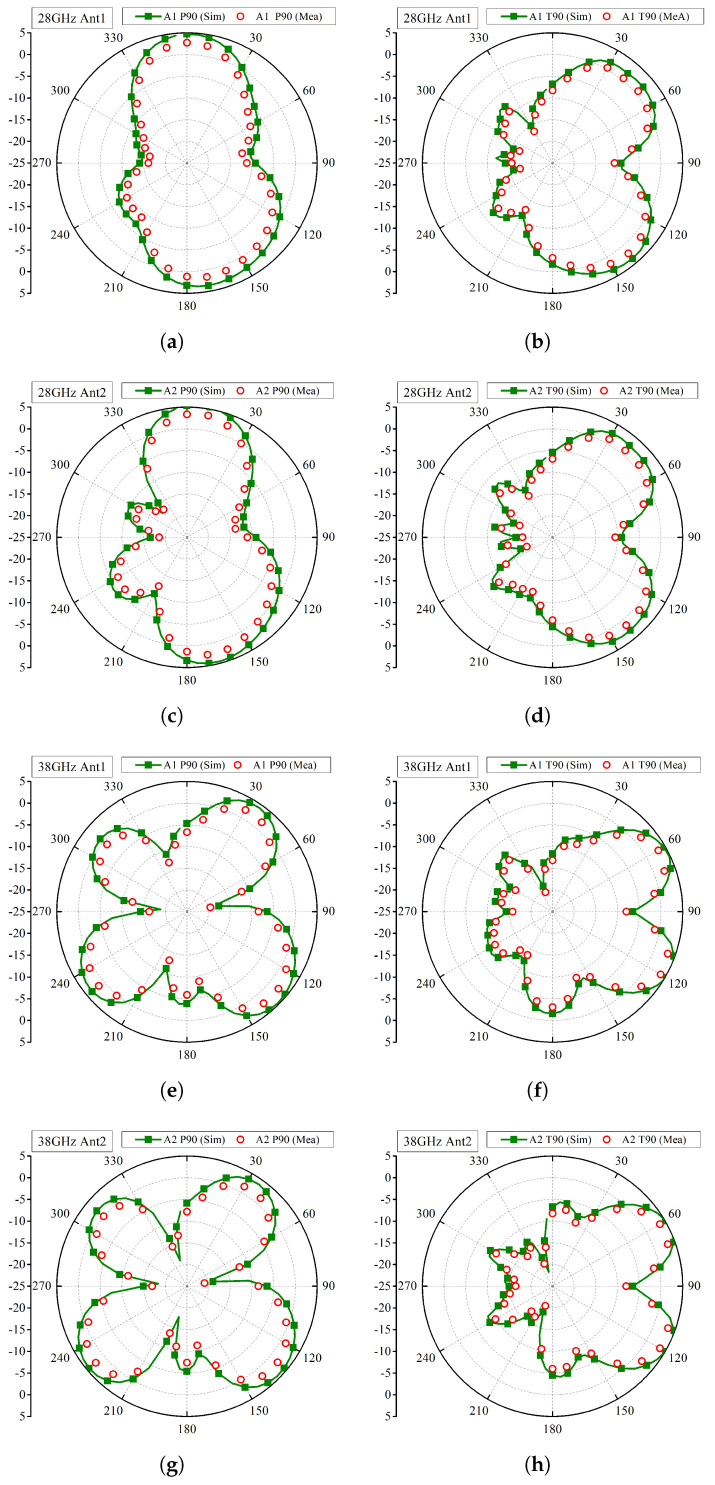
Olympeak MIMO antenna radiation pattern (**a**) Antenna 1 28 GHz Phi 90 (**b**) Antenna 1 28 GHz Theta 90 (**c**) Antenna 2 28 GHz Phi 90 (**d**) Antenna 2 28 GHz Theta 90 (**e**) Antenna 1 38 GHz Phi 90 (**f**) Antenna 1 38 GHz Theta 90 (**g**) Antenna 2 38 GHz Phi 90 (**h**) Antenna 2 38 GHz Theta 90.

**Figure 12:**
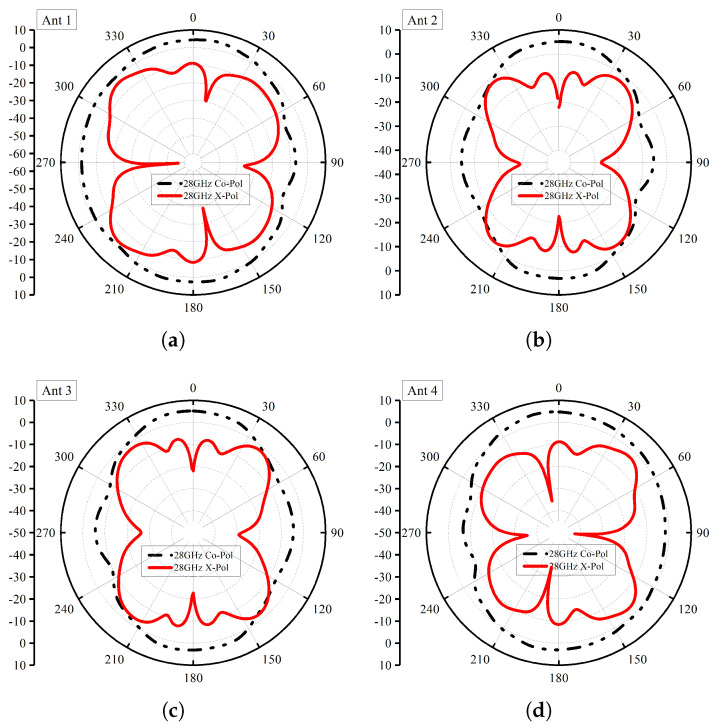
Co- and cross-polarization (**a**) Antenna 1 (**b**) Antenna 2 (**c**) Antenna 3 (**d**) Antenna 4.

The array is symmetric from the center. The radiation patterns of the left two elements are identical to the right two elements in the array. Therefore, analyzing only half of the array would be adequate to obtain an idea for the overall array. We will present the results of Antenna 1 and Antenna 2 when the antennas are sequentially numbered from left to right. The simulated and measured radiation patterns of Antenna 1 and Antenna 2 at two principle planes of Phi = 90 and Theta = 90, at two resonating bands are shown in Figure 11. It is shown from Figure 10a–d that at 28 GHz both Antenna 1 and Antenna 2 have beams on the directions of Theta = 0 and Theta = 180 for Phi = 90, whereas they have main lobes on the directions of Phi = 45 and Phi = 135 for Theta = 90. Likewise, it is shown from Figure 11e–h that at 38 GHz, both Antenna 1 and Antenna 2 have main beams on the directions of Theta = +45 and Theta = 135 for Phi = 90 whereas they have main lobes on the directions of Phi = 60 and Phi = 120 for Theta = 90. The radiation patterns both simulated and measured are in a good agreement with slight disruptions due to the difficulty of thin substrate handling. The co- and cross-polarization of the MIMO system is shown in Figure 12. From the figure, it can be seen that X-pol levels among MIMO elements are well below −10 dB.

## 5. MIMO Performance Parameters

The MIMO performance of the proposed antenna systems were evaluated. These parameters included Enveloper Correlation Coefficient (ECC), Diversity Gain (DG) and Mean Effective Gain (MEG).The Envelope Correlation Coefficient reveals the degree of radiation pattern independence between two antennas. Therefore, the two radiating structures would have a correlation of 0 if one was totally horizontally polarized and the other was completely vertically polarised. The ECC of these antennas would also be zero if one antenna exclusively radiated energy towards the sky and the other just radiated energy towards the earth. Therefore, Envelope Correlation Coefficient considers the polarization, shape, and even the relative phase of the fields between the two antennas of the antennas. The ECC is evaluated using far field characteristics of proposed antenna using Equation (1) [27,28,29]. The ECC among any two radiating elements were found to be <0.005.

**Figure 13:**
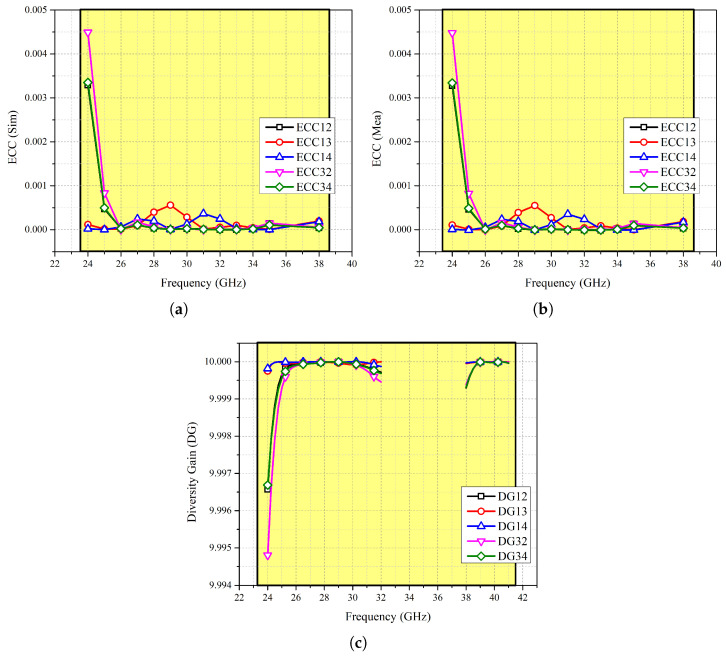
Olympeak antenna MIMO parameters (**a**) ECC Sim (**b**) ECC Mean (**c**) Diversity Gain.

Stand-alone antenna gain is not a trustworthy measure of antenna performance because the antenna is frequently used outside of an anechoic chamber. The antenna is used in a specific situation for a specific application. It is essential to look into how the surroundings influence the antenna’s radiation properties in order to evaluate its performance. The MEG shows how well the antenna will behave in a multi-path environment and is calculated using Equation (2) [30,31,32,33,34]. The diversity gain is calculated using Equation (3). All the evaluated performance parameters results were found to be in adequate limits and thus satisfying the performance criteria for the proposed system. Table 1 shows the MEG of the MIMO Antenna. Also Figure 13 shows the MIMO parameters of the proposed system

(1) $$ECC=\frac{|{\;\int \int \;}_{4\pi }\left(\vec{B_{i}}\left(\theta ,\phi \right)\right)\times \left(\vec{B_{j}}\left(\theta ,\phi \right)\right){\;d\Omega |}^{2}}{{\;\int \int \;}_{4\pi }|\left(\vec{B_{i}}\left(\theta ,\phi \right)\right){|}^{2}\;d\Omega {\;\int \int \;}_{4\pi }|\left(\vec{B_{j}}\left(\theta ,\phi \right)\right){|}^{2}\;d\Omega }$$

where $\vec{B_{i}}\left(\theta ,\phi \right)$ denotes the 3D radiation pattern upon excitation of the *i*-th antenna and $\vec{B_{j}}\left(\theta ,\phi \right)$ denotes the 3D radiation pattern upon excitation of the *j*-th antenna. Ω is the solid angle.

(2) $$MEG=\int_{-\pi }^{\pi }\int_{0}^{\pi }\left[\frac{r}{r+1}G_{\theta }\left(\theta ,\phi \right)P_{\theta }\left(\theta ,\phi \right)+\frac{1}{1+r}G_{\phi }\left(\theta ,\phi \right)P_{\phi }\left(\theta ,\phi \right)\right]sin\theta d\theta d\phi$$

where $G_{\phi }\left(\theta ,\phi \right)$ and $P_{\theta }\left(\theta ,\phi \right)$ are angle of arrival and *r* is the cross-polar ratio which can be expressed as Equation (3).

(3) $$r=10log_{10}\left(\frac{P_{vpa}}{P_{hpa}}\right)$$

The power received by the vertically polarized and horizontally polarized antenna is represented as $P_{vpa}$ and $P_{hpa}$, respectively.

(4) $$DG=10\sqrt{1-(ECC^{2})}$$

Table 2 shows a comparison of the proposed MIMO antenna system with the recent state-of-the-art published literature in terms of the number elements in the system, the frequency coverage of the system, the size of the system, the realized gain, the isolation levels between antenna elements and the Effective Channel Capacity. It is seen from the table that the proposed antenna not only covers the widest bandwidth but also supports dual band operations in the 5G mmwave bands. The isolation performance of the proposed antenna is one of best based on the overall antenna size. The antenna in Ref [7] was the only one which looks close to or better than our proposed antenna with its support of more number of bands and smaller size, however, it has lower gain and its isolation performance and effective channel capacity level were not reported in the article.

## 6. Conclusions

In recent years, 5G technology has emerged as the most significant communication service as the globe moves towards a new era of communication. This is due to the fact that 5G technology provides low latency over Multiple Input Multiple Output (MIMO) fading conditions with high channel capacity, channel aggregation and a higher number of users. On the other hand, new portable gadgets are becoming lighter and thinner, while requiring powerful processing capabilities. This work presented a four-port MIMO antenna system with high performance characteristics. The proposed antenna was shaped in a hybrid Olympic sign, hence it was named as Olympeak antenna. The total unit size of the proposed antenna was 12 × 14 × 0.254 mm${}^{3}$ while for four element MIMO assembly it was noted to be 48 × 14 × 0.254 mm${}^{3}$ (4.48λ${}_{0}$ × 1.12λ${}_{0}$ × 0.02λ${}_{0}$). The antenna was fabricated and tested using in house facilities and the simulated results were found to be in an excellent agreement with the simulations. The gain of the MIMO system at 28 and 38 GHz was noted to be 11 and 10.9 dBi, respectively while the minimum isolation among any two two radiating elements was found to be <20 dB. Through the achieved results, the proposed MIMO system can be considered as potential candidate for future mm-wave applications.

## Figures and Tables

**Table 1:** MEG of the proposed antenna system.

Frequency (GHz)	MEG1	MEG2	MEG3	MEG4
28	−3.13	−3.25	−3.16	−3.88
38	−3.56	−3.77	−3.88	−3.66

**Table 2:** Comparison of the proposed antenna system with the literature.

Ref.	Ant.	Frequency (GHz)	Bandwidth MHz	Size mm${}^{2}$	Gain (dBi)	Isolation (dB)	ECC
[7]	4	23.45–30.79	734/402	13 × 11.25	4.5	NA	NA
		/31.72–35.74	/330				
		/36.3–39.60					
[8]	4	26–31	5	48 × 31	5.2	21	0.0015
[9]	4	26.5–29.5	3	30 × 30	6	>30	0.0005
[10]	4	27.5–28.5	1/1	24 × 24	7	>25	0.001
		/37.5–38.5					
[11]	4	26–30	4	30 × 30	6,12	>25	0.16
[12]	4	26–29.5	350	25 × 15	7.8	17	0.00016
[13]	4	36.83–40.0	317	47.4 × 32.5	6.5	45	0.01
[14]	4	26.64–28.55	191	35 × 35	5.07	27	0.0005
Prop.	4	23–33/37.75–41	10/325	48 × 12	5.7	>20	0.00015

## Data Availability

All the data is available in the study.

## References

[1] Kiani S.H., Marey M., Rafique U., Shah S.I.H., Bashir M.A., Mostafa H., Wong S.W., Parchin N.O. (2022). A Deployable and Cost-Effective Kirigami Antenna for Sub-6 GHz MIMO Applications. Micromachines.

[2] Costanzo S., Venneri F., Massa G.D., Borgia A., Costanzo A., Raffo A. (2016). Fractal Reflectarray Antennas: State of Art and New Opportunities. Int. J. Antennas Propag..

[3] Kiani S.H., Marey M., Savci H.Ş., Mostafa H., Rafique U., Khan M.A. (2022). Dual-Band Multiple-Element MIMO Antenna System for Next-Generation Smartphones. Appl. Sci..

[4] Ibrahim M.S. (2019). Design of low-cost, circularly polarized, and wideband U-slot microstrip patch antenna with parasitic elements for WiGig and WPAN applications. Appl. Comput. Electromagn. Soc..

[5] Sakr A.A., Dyab W.M., Wu K. (2018). A dually polarized six-port junction based on polarization-selective coupling for polarization-inclusive remote sensing. IEEE Trans. Microw. Theory Tech..

[6] Dyab W., Sakr A.A., Wu K. (2018). Millimeter-Wave Polarization-Inclusive Remote Sensing System Based on Dually-Polarized Six-Port Junction. Proceedings of the 2018 11th Global Symposium on Millimeter Waves (GSMM).

[7] Ibrahim M.S., Attia H., Cheng Q., Mahmoud A.A. (2020). Wideband Circularly Polarized Aperture Coupled DRA Array with Sequential-Phase Feed at X-Band.

[8] Wani Z., Abegaonkar M.P., Koul S.K. (2018). A 28-GHz antenna for 5G MIMO applications. Prog. Electromagn. Res. Lett..

[9] Hussain M., Mousa A.E., Jarchavi S.M.R., Zaidi A., Najam A.I., Alotaibi A.A., Althobaiti A., Ghoneim S.S. (2022). Design and Characterization of Compact Broadband Antenna and Its MIMO Configuration for 28 GHz 5G Applications. Electronics.

[10] Raheel K., Altaf A., Waheed A., Kiani S.H., Sehrai D.A., Tubbal F., Raad R. (2021). E-shaped H-slotted dual band mmWave antenna for 5G technology. Electronics.

[11] Kamal M.M., Yang S., Ren X.C., Altaf A., Kiani S.H., Anjum M.R., Saeed S.I. (2021). Infinity shell shaped MIMO antenna array for mm-wave 5G applications. Electronics.

[12] Rahman S., Ren X.C., Altaf A., Irfan M., Abdullah M., Muhammad F., AlKahtani F.S. (2020). Nature inspired MIMO antenna system for future mmWave technologies. Micromachines.

[13] Sehrai D.A., Asif M., Shoaib N., Ibrar M., Jan S., Alibakhshikenari M., Lalbakhsh A., Limiti E. (2021). Compact quad-element high-isolation wideband MIMO antenna for mm-wave applications. Electronics.

[14] Sharma A., Sarkar A., Adhikary M., Biswas A., Akhtar M.J. SIW fed MIMO DRA for future 5G applications. Proceedings of the 2017 IEEE International Symposium on Antennas and Propagation & USNC/URSI National Radio Science Meeting.

[15] Kiani S.H., Alharbi A.G., Khan S., Marey M., Mostafa H., Khan M.A. (2022). Wideband three loop element antenna array for future 5G mmWave devices. IEEE Access.

[16] Iqbal A., ASaraereh O., Bouazizi A., Basir A. (2018). Metamaterial-based highly isolated MIMO antenna for portable wireless applications. Electronics.

[17] Costanzo S., Venneri F., Borgia A., Di Massa G. (2020). Dual-band dual-linear polarization reflectarray for mmWaves/5G applications. IEEE Access.

[18] Buonanno G., Solimene R. (2016). Comparing different schemes for random arrays. Prog. Electromagn. Res. B.

[19] Wang Y., Du Z. (2014). A wideband printed dual-antenna with three neutralization lines for mobile terminals. IEEE Trans. Antennas Propag..

[20] Xing H., Wang X., Gao Z., An X., Zheng H.-X., Wang M., Li E. (2020). Efficient Isolation of an MIMO Antenna Using Defected Ground Structure. Electronics.

[21] Dey S., Dey S., Koul S.K. (2021). Isolation improvement of MIMO antenna using novel EBG and hair-pin shaped DGS at 5G millimeter wave band. IEEE Access.

[22] Zhang C., Chen Z., Shi X., Yang Q., Dong G., Wei X., Liu G. (2022). A Dual-Band Eight-Element MIMO Antenna Array for Future Ultrathin Mobile Terminals. Micromachines.

[23] Xi S., Cai J., Shen L., Li Q., Liu G. (2023). Dual-Band MIMO Antenna with Enhanced Isolation for 5G NR Application. Micromachines.

[24] BharathiDevi B., Kumar J. (2022). Small frequency range discrete bandwidth tunable multiband MIMO antenna for radio/LTE/ISM-2.4 GHz band applications. AEU-Int. J. Electron. Commun..

[25] Nikam P.B., Kumar J., Sivanagaraju V., Baidya A. (2022). Dual-band reconfigurable EBG loaded circular patch MIMO antenna using defected ground structure (DGS) and PIN diode integrated branch-lines (BLs). Measurement.

[26] Roshani S., Shahveisi H. (2022). Mutual Coupling Reduction in Microstrip Patch Antenna Arrays Using Simple Microstrip Resonator. Wirel. Pers. Commun..

[27] Malathi Josephine A.C., Thiripurasundari D. (2016). Review on isolation techniques in MIMO antenna systems. Indian J. Sci. Technol..

[28] Nadeem I., Choi D.Y. (2018). Study on mutual coupling reduction technique for MIMO antennas. IEEE Access.

[29] Alibakhshikenari M., Babaeian F., Virdee B.S., Aïssa S., Azpilicueta L., See C.H., Althuwayb A.A., Huynen I., Abd-Alhameed R.A., Falcone F. (2020). A comprehensive survey on “Various decoupling mechanisms with focus on metamaterial and metasurface principles applicable to SAR and MIMO antenna systems”. IEEE Access.

[30] Zhang S., Ying Z., Xiong J., He S. (2009). Ultrawideband MIMO/diversity antennas with a tree-like structure to enhance wideband isolation. IEEE Antennas Wirel. Propag. Lett..

[31] Cuneray K., Akcam N., Okan T., Arican G.O. (2023). 28/38 GHz dual-band MIMO antenna with wideband and high gain properties for 5G applications. AEU-Int. J. Electron. Commun..

[32] Kuzu S., Akcam N. (2016). Array antenna using defected ground structure shaped with fractal form generated by Apollonius circle. IEEE Antennas Wirel. Propag. Lett..

[33] Govindan T., Palaniswamy S.K., Kanagasabai M., Kumar S., Marey M., Mostafa H. (2022). Design and analysis of a flexible smart apparel MIMO antenna for bio-healthcare applications. Micromachines.

[34] Ravi K.C., Kumar J. (2022). Miniaturized Parasitic Loaded High-Isolation MIMO Antenna for 5G Applications. Sensors.

